# Cortical Tracking of Sung Speech in Adults vs Infants: A Developmental Analysis

**DOI:** 10.3389/fnins.2022.842447

**Published:** 2022-04-12

**Authors:** Adam Attaheri, Dimitris Panayiotou, Alessia Phillips, Áine Ní Choisdealbha, Giovanni M. Di Liberto, Sinead Rocha, Perrine Brusini, Natasha Mead, Sheila Flanagan, Helen Olawole-Scott, Usha Goswami

**Affiliations:** ^1^Department of Psychology, Centre for Neuroscience in Education, University of Cambridge, Cambridge, United Kingdom; ^2^School of Computer Science and Statistics, Trinity College Dublin, Dublin, Ireland; ^3^Laboratoire des Systèmes Perceptifs, UMR 8248, CNRS, Ecole Normale Supérieure, PSL Research University, Paris, France; ^4^Institute of Population Health, University of Liverpool, Liverpool, United Kingdom

**Keywords:** EEG, language, neural oscillations, TRF, cortical tracking

## Abstract

Here we duplicate a neural tracking paradigm, previously published with infants (aged 4 to 11 months), with adult participants, in order to explore potential developmental similarities and differences in entrainment. Adults listened and watched passively as nursery rhymes were sung or chanted in infant-directed speech. Whole-head EEG (128 channels) was recorded, and cortical tracking of the sung speech in the delta (0.5–4 Hz), theta (4–8 Hz) and alpha (8–12 Hz) frequency bands was computed using linear decoders (multivariate Temporal Response Function models, mTRFs). Phase-amplitude coupling (PAC) was also computed to assess whether delta and theta phases temporally organize higher-frequency amplitudes for adults in the same pattern as found in the infant brain. Similar to previous infant participants, the adults showed significant cortical tracking of the sung speech in both delta and theta bands. However, the frequencies associated with peaks in stimulus-induced spectral power (PSD) in the two populations were different. PAC was also different in the adults compared to the infants. PAC was stronger for theta- versus delta- driven coupling in adults but was equal for delta- versus theta-driven coupling in infants. Adults also showed a stimulus-induced increase in low alpha power that was absent in infants. This may suggest adult recruitment of other cognitive processes, possibly related to comprehension or attention. The comparative data suggest that while infant and adult brains utilize essentially the same cortical mechanisms to track linguistic input, the operation of and interplay between these mechanisms may change with age and language experience.

## Introduction

The amplitude envelope of speech carries crucial low-frequency acoustic information that assists linguistic decoding at multiple time scales. According to “multi-time resolution” models of speech perception, linguistic decoding is achieved in part via the neural tracking of different temporal modulation patterns in speech at different timescales simultaneously (Poeppel, 2003; Greenberg, 2006; Luo and Poeppel, 2007; Ghitza and Greenberg, 2009; Chait et al., 2015). One core mechanistic proposal is that cortical oscillations entrain or phase-align their activity to modulations at corresponding timescales in the signal, thereby encoding the different energy patterns, and binding the information together into the final speech percept (Ghitza, 2011, 2012; Giraud and Poeppel, 2012; Poeppel, 2014). Neurophysiological signals are known to preferentially track the amplitude envelope of adult-directed speech (ADS) in the theta band, approximately 4–8 Hz (Luo and Poeppel, 2007; Ghitza, 2011; Ding and Simon, 2013, 2014; Gross et al., 2013; Zion Golumbic et al., 2013; Doelling et al., 2014; Di Liberto et al., 2015; Kösem and van Wassenhove, 2017). However, recent analyses of neural tracking of the speech amplitude envelope by infants shows preferential tracking in the delta band [∼0.5–4 Hz, see Attaheri et al. (2022)]. This developmental difference (infants primarily engage delta, adults primarily engage theta) may reflect the nature of the stimulus, as acoustic analyses of infant-directed speech (IDS) using a spectral-amplitude modulation phase hierarchy approach (S-AMPH, see Leong et al., 2017) reveal significantly greater modulation energy compared to ADS in an amplitude-modulation (AM) band centered on ∼2 Hz. Attaheri et al. (2022) were the first to compare infant cortical tracking in the delta and theta bands, as prior infant cortical tracking studies relied on the broadband speech envelope (Kalashnikova et al., 2018; Jessen et al., 2019). Accordingly, it could be argued that cortical tracking of IDS by delta-band neural signals may be key to initial language acquisition.

An outstanding question is whether the preferential delta-band tracking observed with infants (Attaheri et al., 2022) was driven by the IDS stimulus materials, and accordingly whether the adult brain would show similar preferential delta-band tracking to the same speech input. Note that delta-band tracking of speech input was also observed in the adult brain in studies that did not use IDS (Molinaro et al., 2016; sentences; Di Liberto et al., 2015, 2018; story listening, Kösem and van Wassenhove, 2017, for review). In order to ensure infant attention and engagement, Attaheri et al. (2022) used videos of a female singing nursery rhymes in a deliberately infant-directed manner (IDS), recording neural responses to this audio-visual input using 64-channel EEG. Nursery rhymes are inherently rhythmic, and neural responses in musical tasks are also typically strong in the delta band (Cirelli et al., 2016). Thus, the preferential delta-band tracking observed in the infants studied by Attaheri et al. (2022) might either reflect the use of IDS, or the fact that the speech input was sung, or both. Adult entrainment data may help to disentangle some of these possibilities, as the adult brain may respond differently to IDS compared to the infant brain.

Accordingly, here we report a replication of Attaheri et al. (2022) with adults. Identical stimuli, identical experimental procedures and identical analysis pipelines (bar removal of adult ocular artifacts not present in the infant data; see section “Materials and Methods” for full details) were adopted to allow a faithful comparison of neural tracking of sung speech in the infant versus fully-matured adult brain. Speech also contains acoustic information within its higher-frequency bands (beta, gamma) that is linguistically important. Low-frequency (delta, theta) phase and high-frequency (gamma) amplitudes track acoustic rhythms in the adult brain by operating together as an integrated representational mechanism, called phase-amplitude coupling, PAC (Gross et al., 2013; Hyafil et al., 2015; Lizarazu et al., 2019). In the adult brain, strongest PAC is observed for theta-gamma coupling (Gross et al., 2013). Attaheri et al. (2022) demonstrated PAC in infants at all ages studied (4, 7, and 11 months). However, they reported that delta acted as an equally strong carrier phase for higher-frequency amplitudes (beta and gamma) as theta phase. For infants, therefore, both delta and theta play important roles in the temporal organization of higher-frequency amplitudes during speech processing. Studying PAC to the same sung speech in adults enables clarification of whether the PAC previously observed with infants is stimulus-driven or development-driven.

On a stimulus-driven hypothesis, delta-based PAC should be as strong as theta-based coupling for adults when sung speech/IDS is the input. Alternatively, PAC in the adult brain may always favor theta-gamma organization. Also of interest was whether low-frequency phases (delta and theta) would act as equally strong carriers for both beta and gamma band information, as found for infants, or whether coupling to beta would be less important for adults (Hyafil, 2017). Attaheri et al. (2022) reported that whilst both gamma and beta high-frequency amplitudes coupled to delta and theta phases, it was amplitudes in the gamma band that produced the stronger coupling to both delta and theta phases in the infants. This effect did not change between 4 and 11 months of age. Again, it is currently unclear whether this is a developmental or a stimulus-driven phenomenon.

Theoretically, one key difference between adults and infants, when listening to the same speech input, is the accumulated years of prior experience with speech processing enjoyed by the adults. Infants are universal novices, and even by 11 months, are unlikely to yet comprehend the different nursery rhymes that they are hearing. They also cannot yet produce recognizable speech. In adult neural speech processing studies, delta-band tracking is frequently related to discourse-level parsing related to phrasing (Ding et al., 2016; Kösem and van Wassenhove, 2017), as well as to auditory attentional mechanisms linked to the automatic grouping of sounds (Boucher et al., 2019). While the pre-verbal infant brain may also utilize acoustic grouping mechanisms, which could be stimulus-driven, infants are unlikely to have learned discourse-level speech information by 11 months. Similarly, in adult studies theta band cortical tracking has been related to syllable parsing and speech intelligibility (Luo and Poeppel, 2007; Peelle et al., 2013; Millman et al., 2015; Baltzell et al., 2017). While there may be stimulus-driven cues for syllable parsing that infants can utilize automatically (such as the acoustic edges linked to syllable rise times, see Doelling et al., 2014; Lizarazu et al., 2021), speech intelligibility is unlikely to be a key factor in cortical tracking in infancy, as infants are still learning spoken language. Indeed, animal studies have also demonstrated cortical tracking of rhythmically-structured acoustic input in both delta and theta bands, possibly suggesting that these mechanisms are triggered by general auditory perceptual abilities conserved across mammalian species (Lakatos et al., 2005, 2016). The current study should throw light on these important developmental questions.

## Materials and Methods

The materials and methods used in the current analysis replicated those used in a prior study with infants (Attaheri et al., 2022). Any subtle adaptions required in adapting the experiment for adult participants are outlined below.

### Participants

Total of 22 monolingual, English-speaking, participants (11M, 11F, aged 18–30, Mean age 21 years) were recruited from central Cambridge (United Kingdom) and surrounding areas. The study was reviewed by the Psychology Research Ethics Committee of the University of Cambridge and after a detailed explanation of the study, written consent was given by each participant. Each participant reported no history of language difficulties or dyslexia. One participant’s data was excluded due to a technical error (no stimulus triggers were recorded) leaving 21 participants data remaining for analysis.

### Stimuli

A selection of 18 typical English language nursery rhymes were chosen as the stimuli. Audio-visual stimuli of a singing head were recorded using a Canon XA20 video camera at 1,080p, 50fps and with audio at 4,800 Hz. A native female speaker of British English used infant directed speech to melodically sing (for example “Mary Mary Quite Contrary”) or rhythmically chant (for nursery rhymes like “There was an old woman who lived in a shoe”) the nursery rhymes whilst listening to a 120 BPM metronome. The beat was not present on the stimuli presented to the participants, but it ensured that a consistent quasi-rhythmic production was maintained throughout the 18 nursery rhymes. During recording of the video, the adult was singing to a real infant, with whom she was sharing mutual gaze.

### EEG Data Collection

Participants were seated ∼650 mm away from the presentation screen within a sound-proof acoustic chamber. EEG data was recorded at a sampling rate of 1,000 Hz using a GES 300 amplifier connected to a correctly sized 128 channel electrode net (Geodesic Sensor Net, Electrical Geodesics Inc., Eugene, OR, United States). The sounds were presented at 60 dB (dBA, checked by a handheld sound level meter) from speakers (Q acoustics 2020i driven by a Cambridge Audio Topaz AM5 Stereo amplifier) placed either side of the screen. Participants were asked to attend to the screen whilst 18 nursery rhyme videos played sequentially, each repeated three times (54 videos, with a presentation time of 20′ 33′′ in total). All participants included in analysis completed the full experiment. The stimulus period was followed by a 5 min resting state recording, in which the participants were asked to sit silently with their eyes open, whilst no sound or visual stimuli were present.

### EEG Preprocessing

All analyses were conducted with custom-made scripts in Matlab 2017a (The MathWorks, Inc., Natick, MA, United States) incorporating the EEGLab toolbox (Delorme and Makeig, 2004). The analysis protocols were kept as consistent as possible to the previous infant analysis pipelines to allow faithful comparison to the previously reported infant results.

The EEG data, from the 128 channels, was first filtered (*pop_eegfiltnew* function of EEGLab toolbox) into a broadband signal (0.5–45 Hz) using zero-phase bandpass Hamming windowed FIR filters (transition band widths of 2 Hz with cutoff frequencies at −6 dB). The EEG data was down sampled to 100 Hz to reduce the computational load. Next, the *clean_asr* EEGLab function (Delorme and Makeig, 2004) was used to clean noise artifacts from the data by identifying and removing bad principal components *via* a modified PCA procedure (see Supplementary Material for more details). Further bad channels were identified *via* probability and kurtosis and were interpolated (*via* spherical interpolation), if they were 3 standard deviations away from the mean, before all channels were re-referenced to a 128-channel average reference. ICA (*runica*; EEGLab) was conducted to detect components containing ocular and ECG artifacts, which were visually identified and then removed from the data. Frequency bands of interest (0.5–4 Hz, 4–8 Hz or 8–12 Hz for the mTRF analysis) were acquired using a using zero-phase bandpass Hamming windowed FIR filters (transition band widths of 2 Hz with cutoff frequencies at −6 dB, 0–5 Hz, 3–9 Hz and 7–13 Hz, respectively).

EEG responses were epoched into trials aligned to the start and ending at the completion of a phrase (e.g., “Mary had a little lamb”), producing EEG responses to 83 phrases (*M length* ± *SD*: 4.23 s ± 0.88) which were repeated a maximum of 3 times in the experiment (249 epochs in total). This epoching procedure was used to keep consistency with the previous infant EEG study. To retain epochs where a single channel exhibited noise epoch by epoch channel interpolations were conducted. Per epoch, probability and kurtosis were used to identify bad channels and were interpolated (*via* spherical interpolation) if they were 3*SD* away from the mean.

#### Multivariate Temporal Response Function

TRFs are encoding models that can describe how an input and output of a system are related *via* linear convolution (Crosse et al., 2016). Here, we applied TRFs in a backward direction to assess how strongly a stimulus property, in this case the stimulus envelope, is encoded in the neural response. We chose backward TRF modeling as it uses information from all EEG channels at once to reconstruct the speech envelope, giving a low weighting to irrelevant channels whilst allowing the model to capture additional variance across channels (Crosse et al., 2016). The result is a single objective metric (i.e., the envelope decoding correlation). Backward TRF modeling has the advantage of producing larger correlation scores compared to forward TRF modeling, making it a good choice for analyzing the original infant EEG data, which is inherently noisy (Jessen et al., 2021).

After preprocessing, the epochs of EEG data in response to each nursery rhyme trial were averaged together to improve the signal to noise ratio of the data for the mTRF analysis (matching the infant analysis procedure). The mTRF analysis was conducted using the multivariate temporal response function (mTRF) toolbox v1.5 (Crosse et al., 2016) through Matlab 2017a (The MathWorks, Inc., Natick, MA, United States). The backward model can be expressed by the following formula in which the reconstructed stimulus envelope *s(t)* is created by a linear decoder, g*(τ*, n), mapping the neural response, *r(t,n)*, back to the stimulus, *s(t)*. The TRF decoder was used to integrate the neural responses at multiple time lags, τ, between 0 and 250 ms (τ_*min*_ = 0 ms, τ_*max*_ = 250 ms). These “stimulus-relevant” time lags where selected in keeping with the previous literature (Ding and Simon, 2014; Di Liberto et al., 2015; Crosse et al., 2016).


$$\hat{\text{s}}\,\left(t\right)=\sum_{n}\sum_{\tau }r\,\left(t+\tau ,n\right)\,g\,\left(\tau ,n\right),$$

The quality of the envelope tracking within each EEG frequency band was assessed by a “leave-one-out” cross-validation per participant. First the average trial EEG epochs (maximum of 83) were normalized *via* function *nt_normcol* (Noisetools^1^). Normalization, decreased the range of values that were necessary for the regularization parameter search in the mTRF toolbox, making the cross validation more efficient. Next, the normalized epoch trials were rotated M-1 times, each serving once as the “test set” with the remainder of the trials being the TRF “training set.” For each rotation, the resultant M-1 training models were averaged to create one average model from the training set. The average model was subsequently convolved with the test data to reconstruct the stimulus. Pearson’s correlation (r) was used to validate how well the reconstructed stimulus correlated to the original. This process was repeated for the M-1 rotations. To avoid overfitting the model to a specific trial, an average *r* value was taken from the 83 r validation values. This process was repeated at 12 ridge regressions (λ values, 1 × 10^–3^:1 × 10^8^) with the lowest λ value, where any increase gave no further improvement to the average *r* value, was taken (Crosse et al., 2016). Choosing the correct lambda value here again mitigated the potential overfitting of the TRF model. This average *r* value, at the optimal λ, was used for all further analysis.

#### mTRF Auditory Stimuli Preprocessing

The envelope of the auditory signal was extracted by taking the absolute value of the analytic signal generated by the Hilbert transform (Matlab). As the envelope of the lower frequencies is linearly relatable to the EEG signal (Pasley et al., 2012; Zion Golumbic et al., 2013; O’Sullivan et al., 2015) the envelope of the stimuli was filtered between 0.5 and 15 Hz (lowpass; 6th order Butterworth filter. Highpass; 9th order Butterworth filter). The resultant envelopes were normalized using *nt_normcol* (NoiseTools^1^). Finally, the stimulus envelopes were down-sampled to 100 Hz to match the EEG signal.

#### mTRF Random Permutation Statistics

Random permutation statistics were created for each participant to measure the average stimulus reconstruction (r) that could be obtained by chance. The random permutation procedure was conducted per participant for each frequency band producing a paired chance stimulus reconstruction (r). To obtain a random permutation of the data, whilst maintaining phase integrity, each of the stimulus envelopes were first reversed and a random circular shift was applied. Next, the mTRF cross-validation was ran in the same way as the real data (see above for details), to give a stimulus reconstruction (r) value. This procedure was iterated 100 times to create a null distribution and the average of these 100 iterations were used as that participant’s random stimulus reconstruction (r) value.

### Spectral Analysis (Periodogram Power Spectral Density Estimate)

All remaining epochs after preprocessing were concatenated back into one continual EEG signal. A one-sided PSD estimate was conducted separately for each electrode channel using the periodogram function (Matlab). The length of the participants data was zero padded to ensure the size of the rectangular window used was equal in length to the number of discrete Fourier transform (DFT) points, ensuring the correct FFT across participants. This resulted in 52,834 equal spaced frequency bins from 0 to 50 Hz.

The periodogram can be defined by the following formula. In which the EEG signal, *x*_*n*_, is sampled at 100Hz, with Δ*t* as the sampling interval.


$$\hat{P}\,\left(f\right)=\frac{\Delta \,t}{N}\,\left|\sum_{n=0}^{N-1}x_{n}\,e^{-j\,2\,\pi \,f\,\Delta \,t\,n}\right|\,\begin{array}{c} 2 \end{array},-1/2\,\Delta \,t<f\leq 1/2\,\Delta \,t,$$

**FIGURE 1:**
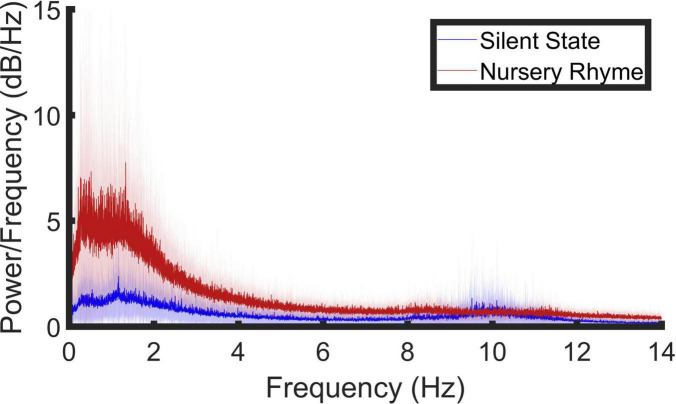
Spectral decomposition of the EEG signal (0.5–14 Hz) in response to nursery rhyme stimulation. A periodogram was used to obtain a power spectral density (PSD) estimate separately for the resting state (blue line) and the nursery rhyme stimulus (red line) periods. Bold lines indicate the mean values and pale shading plots the standard deviation of the data. Outlier analysis was also conducted to remove extreme data points leaving, resting state, *N* = 17; Nursery Rhyme stimulus period *N* = 17.

To achieve the one-sided periodogram output reported in Figure 1, values at all frequencies (except 0 and the Nyquist, 1/2Δt), were multiplied by two to conserve the total power.

### Phase Amplitude Coupling

The same concatenated data sets created for the PSD analysis were also used for the Phase Amplitude Coupling (PAC) analysis. A modified version of the WinPACT plugin (EEGLab) (Delorme and Makeig, 2004) was used to acquire normalized modulation index (nMI) values (Özkurt and Schnitzler, 2011), a measure adapted from Canolty et al.’s (2006) modulation index (MI) (Canolty et al., 2006). The normalized version of the MI calculation (nMI) was selected as differences in low-frequency power have been shown to adversely affect the PAC calculation (Canolty et al., 2006; Özkurt and Schnitzler, 2011; Aru et al., 2015). The MI method combines the amplitude envelope time series A1(t + τ) of a high-frequency with the phase time series φ2(t) of a specified low-frequency, creating a composite complex-valued signal z(t, τ). The resulting value is a widely validated metric of the coupling strength and preferred phase between two frequencies. For each participant’s data, low-frequency phase (LFP) and high-frequency amplitude (HFA) were extracted with a zero-phase FIR filter (Hamming window), separately for all 128 electrodes. LFP center frequencies were filtered from 2 to 8 Hz, in 1 Hz steps with a 2 Hz bandwidth, and HFA center frequencies were filtered from 17.5 to 42.5 Hz, in 5 Hz steps with a 5 Hz bandwidth. A sliding 5 s analysis window was implemented, with 2.5 s overlaps, with a mean vector length calculated per window. Next, 200 surrogate statistical iterations were created for each PAC calculation window. The statistically normalized MI estimate was obtained for each analysis window by subtracting the mean and dividing by the standard deviation obtained from a Gaussian fit of surrogate MI estimates (nMI = (Canolty’s MI—surrogate MI Mean) / surrogate MI Std). This statistical procedure was first suggested by Canolty et al. (2006) and implemented in the winPACT plugin based on code adapted from Özkurt and Schnitzler (2011). Each iteration of the surrogate data was created by shuffling the high-frequency amplitude time series *via* circular rotation. A nMI estimate was obtained for each of the 200 surrogate data iterations, from which a 95% confidence interval was calculated using normcdf.m. This step accounted for the mean and standard deviation of the surrogate data set, thus creating an appropriate threshold for the frequency band analyzed (see winPACT_precompute.m subscript in the winPACT toolbox for complete code, implemented in our analysis script). Finally, generalized family-wise error rate correction was implemented to correct for the multiple PAC calculation windows. The remaining statistically significant nMI windows were averaged per channel for each of the PAC pairs (i.e., each LFP and HFA step) separately for each participant. The frequency bands of interest were defined as follows, delta 2–4 Hz, theta 4–8 Hz, beta 15–30 Hz and gamma 30–45 Hz). The channel exhibiting the strongest nMI, within predefined phase and amplitude band groupings (delta/beta, delta/gamma, theta/beta, theta/gamma), was taken forward for the LMEM and for the group level grand average plots.

## Results

The detailed analyses outlined in the Methods were aimed firstly at identifying peaks where the nursery rhymes induced increases in EEG spectral power. The second step was to investigate whether the observed oscillations in the lower frequency bands tracked the envelope of speech. Finally, we were interested to see to what extent the phase of these low frequency (delta and theta) oscillations coupled to the amplitudes of the higher frequency oscillations (beta and gamma).

### Power Spectral Density Response to Resting State and Stimulus Periods

The distribution of low-frequency neural signals within our data was established using spectral decomposition of the signal, achieved using the periodogram power spectral density (PSD) estimate (Figure 1). After preprocessing, PSD was obtained for each of the remaining 128 electrodes in response to audio-visually presented nursery rhymes and during a 5-min period of silence. A grand average across both conditions (stimulus and resting state) and all channels revealed three prominent frequency peaks centered around ∼1.25, ∼8.54, and ∼10.04 Hz (Supplementary Figure 1). The peak visible in Figure 1 at 0.5 Hz was not considered for analysis as its location at the broadband filter boundary means we are unable to discount its occurrence due to a filtering artifact. Due to the prevalence of low-frequency noise in some of the recording sessions, outlier analysis (*isoutlier “quartiles”* function, Matlab) was conducted to remove extreme data points. Four participant’s data points were identified as outliers and removed from the PSD analysis leaving 17 PSD analysis data sets.

To establish whether the PSD peaks in the nursery rhyme stimulus period (NR) were significantly different to the resting state (RS) we used a repeated measures ANOVA. The ANOVA had 2 levels, a level of “condition” (NR versus RS) and a level of “band” (∼1.25, ∼8.54, and ∼10.04 Hz). The band level was included to investigate whether any RS-NR difference was significantly higher throughout all the observed peaks or not. The dependent variable was the maximum PSD value, averaged across channels, taken per participant from a 1 Hz window centered around 1.25, 8.54, and 10.04 Hz, for both the stimulus period and the resting state. Greenhouse-Geisser corrected results showed statistically significant main effects of condition [*F*(1, 16) = 24.372, *p* = 1.49 × 10^–4^], and band; [*F*(1.140,18.239) = 17.046, *p* = 4.2 × 10^–4^] and a significant interaction between condition and peak of the PSD values [*F*(1.112, 17.797) = 18.308, *p* = 3.336 × 10^–4^]. Due to the significant interaction, *post hoc*, Bonferroni corrected, simple main effects analysis was conducted and showed that the stimulus induced PSD was significantly larger than the corresponding peaks in the resting state at 1.25 Hz [*F*(1, 16) = 22.427, *p* = 2.24 × 10^–4^; mean ± SEM, RS = 4.454 ± 0.952, NR = 15.990 ± 3.123] and 8.54 Hz [*F*(1, 16) = 7.791, *p* = 0.013; mean ± SEM, RS = 1.671 ± 0.351, NR = 2.984 ± 0.482] but not at 10.04 Hz [*F*(1, 16) = 0.904, *p* = 0. 356; mean ± SEM, RS = 3.081 ± 0.870, NR = 2.364 ± 0.273].

In summary, the data show stimulus-induced PSD at ∼1.25 and ∼8.54 Hz. A further peak was observed at 10.04 Hz, however, this was not significantly different from resting state.

### Power Spectral Density Comparison to Infant Experiment

No peaks in the PSD spectrum were observed at the corresponding peak frequencies reported in the infant analysis (2.20 and 4.37 Hz). This may imply that the infant neural response was more stimulus-driven, as there were clear modulation peaks in the modulation spectrum of the nursery rhymes at these two frequencies (please see Supplementary Figures 2–4). To compare whether the nursery rhyme stimuli induced power increases in the same regions in the adult EEG data as in the infant EEG data, a two-way repeated measures ANOVA was conducted using the infant PSD peak values as the dependent variable. The maximum PSD value was taken per participant in a 1 Hz window centered around 2.20Hz and 4.37Hz for both the stimulus period and the resting state (band × condition). A statistically significant main effect of condition [*F*(1, 16) = 41.601, *p* = 8.0 × 10^–6^], band; [*F*(1,16) = 24.498, *p* = 1.45 × 10^–4^] and a significant interaction between condition and peak of the PSD values [*F*(1, 16) = 17.230, *p* = 7.52 × 10^–4^] was observed. Due to the significant interaction, *post hoc*, Bonferroni corrected, simple main effects analysis was conducted. This showed that the nursery rhyme stimuli induced a PSD increase from resting state at both ∼2.20Hz [*F*(1, 16) = 29.453, *p* = 5.60 × 10^−5^; mean ± SEM, RS = 3.241 ± 0.696, NR = 10.839 ± 1.771) and ∼4.37 Hz [*F*(1, 16) = 94.241, *p* = 4.14 × 10^–8^; mean ± SEM, RS = 1.636 ± 0.410, NR = 3.741 ± 0.495] in the adult data. Accordingly, although no visible peaks in activity were observed (see Figure 1), significant stimulus-related PSD power was present in the adult data at the matched infant frequencies.

### Delta and Theta EEG Frequency Bands Track Nursery Rhyme Envelopes

**FIGURE 2:**
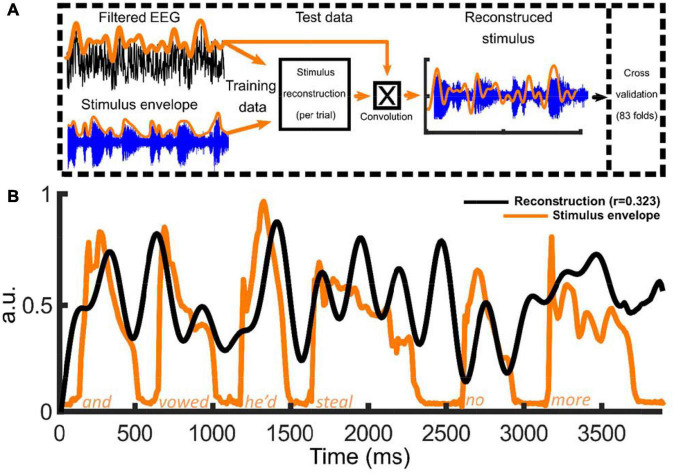
Overview of method to reconstruct the low frequency stimulus envelopes in nursery rhyme phrases using the multivariate temporal response function (mTRF) approach. Panel **(A)** provides a schematic of the stimulus reconstruction model along with a summary of the mTRF analysis pipeline. The EEG signal and the stimulus envelope (absolute value of the Hilbert envelope) were submitted to the mTRF stimulus reconstruction. For the cross validation procedure, 83 nursery rhyme trials were rotated M-1 times each serving once as the “test set” with the remainder of the trials being the “training set.” The process was repeated at 12 lambda values (λ values, 1 × 10^– 3^:1 × 10^8^) with the average model convolved with the test data to reconstruct the stimulus envelope at the optimal λ. Panel **(B)** Example of one of the 83 mTRF stimulus reconstructions (this example trained on 0.5:4 Hz data) for one participant along with the original acoustic stimulus envelope. The black line depicts the reconstruction (in arbitrary units; a.u.) and the orange line illustrating the absolute value of the Hilbert envelope of the nursery rhyme phrase, *“and vowed he’d steal no more”* (in arbitrary units; a.u.).

To investigate the presence and strength of cortical tracking, backward mTRFs (Figure 2A) were employed. The models were trained with either delta (0.5–4 Hz), theta (4–8 Hz) or alpha (8–12 Hz) EEG signals extracted from the EEG recorded to the nursery rhymes. The quality of the stimulus reconstruction was then compared to randomly permuted data (see section “Materials and Methods”). To recap briefly, a backward TRF decoding model was fit separately to the Hilbert envelope of each of the 83 nursery rhyme trials separately for each participant, using a leave-one-out cross-validation procedure (see section “Materials and Methods”). Pearson’s correlation (r) was used to test the quality of the reconstruction (Figure 2B) providing an objective metric of envelope tracking at the individual level. To test the correlation (r) values against chance, random permutation statistics were created for each participant (*N* = 100 permutations).

To examine whether significant cortical tracking was present in each band, a two-way repeated measures ANOVA was employed. This utilized the factors of condition (real mTRF *r* values vs randomly permuted mTRF *r* values) and band (whether these values differed significantly between the analysis bands of 0.5–4 Hz, 4–8 Hz, 8–12 Hz). the data (Greenhouse-Geisser corrected) showed significant main effects of condition [*F*(1, 20) = 20.481, *p* = 2.063 × 10^–4^], band; [*F*(1.250, 24.992) = 53.748, *p* = 2.47 × 10^–8^] and a significant interaction between condition and band [*F*(1.267, 25.340) = 7.772, *p* = 0.007]. Due to the significant interaction, *post hoc*, Bonferroni corrected, simple main effects analysis was employed. This showed that the real mTRF *r* values were significantly larger than chance level (randomly permuted mTRF *r* value) in delta [*F*(1, 20) = 12.900, *p* = 0.0005; mean ± SEM, Rand = 0.019 ± 0.0001, Real = 0.037 ± 0.0003] and theta [*F*(1, 20) = 21.171, *p* = 0.0036; mean ± SEM, Rand = 0.012 ± 0.0001, Real = 0.022 ± 0.002] bands. The alpha band showed a trend toward significance (p = 0.0656), but fell outside our chosen alpha level of *p* = 0.05, [*F*(1, 20) = 6.212, *p* = 0.0656; mean ± SEM, Rand = 0.006 ± 0.0001, Real = 0.009 ± 0.003].

**FIGURE 3:**
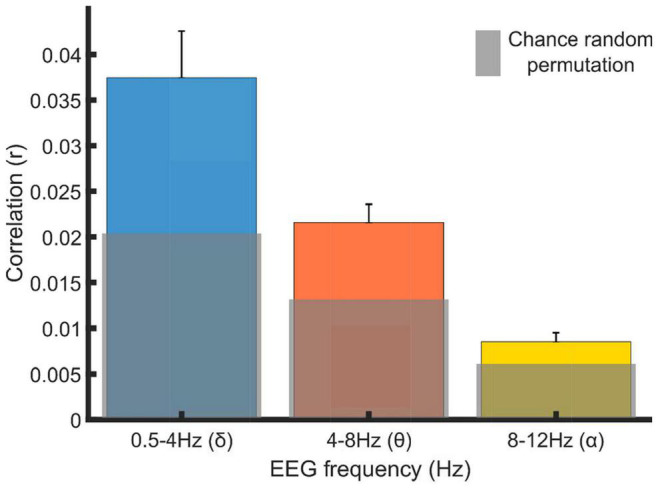
Grand average correlations between stimulus envelope waveforms and their stimulus reconstructions generated by the EEG data from selected frequency bands. Blue, orange and yellow bars show the average correlation value (Pearson’s r; mTRF correlation) and standard error, across the 21 participants. The gray bars show the average random permutation *r* values within each frequency band.

Bayesian, related samples *t*-test, statistics were also conducted to further investigate the relative effect size within each frequency band. This approach enables a stronger test of the alpha band result, as Bayes Factors indicate the strength of evidence for rejecting the null hypothesis. The Bayesian analyses showed that there was very strong evidence for above chance delta cortical tracking (BF10 = 57.84, so the evidence for the “significant tracking” hypothesis is fifty seven times stronger than the evidence for the null hypothesis), decisive evidence for above chance theta cortical tracking (BF10 = 987.54) but only moderate evidence for above chance alpha cortical tracking (BF10 = 3.31; in Bayesian terms this value means that we have only moderate to anecdotal evidence to reject the null hypothesis). Taken together the adult stimulus reconstruction analyses showed that cortical delta and theta neural signals significantly tracked the envelopes of the nursery rhyme stimuli (Figure 3), but there was only moderate evidence for alpha tracking.

A Bonferroni corrected, simple main effects analysis showed that the real mTRF *r* values, in all bands, were significantly different to each other band (Table 1). The finding that delta band values were significantly greater than values in the theta band matches the infant data. However, alpha tracking was not significant in the infant data nor in the adult data, although it approached significance in the latter (*p* = 0.0656), as shown in Figure 3.

**TABLE 1:** Pairwise differences between the mTRF *r* values within each frequency band.

*0.01 **0.00001	*Delta*	*Theta*	*Alpha*
Delta		−0.016*	−0.029**
Theta	0.016*		−0.13**
Alpha	0.029**	0.13**	

*The numbers show the relative difference between the cortical tracking values (r value) between the different frequency bands column heading, in white text, minus the row heading, in black text).*

Bonferroni corrected p values are denoted by *0.01 and **0.00001.

Accordingly, the mTRF stimulus reconstruction data broadly match the results found with infants using the same experimental procedure and stimulus reconstruction analyses (Attaheri et al., 2022). In both infants and adults, delta and theta EEG responses track the acoustic envelope of sung speech. Regarding the differences between infants and adults observed for the alpha band, it is notable that the stimulus-induced PSD peak at 8.54 Hz only shows anecdotal evidence of a mechanistic role in tracking the envelope of the sung speech. Accordingly, the observed increase in low alpha power (Figure 1) may be linked to increased attentional processing (Pichora-Fuller et al., 2016; Dimitrijevic et al., 2017), the recruitment of other language-related processes by the adults (Weisz et al., 2011), or other factors. It is also interesting that the Bayesian analyses run for the adult data showed strong evidence for delta band cortical tracking (BF10 = 20.00) but decisive (i.e., extremely strong) evidence for theta band cortical tracking (BF10 = 166.67). This may imply that theta band tracking increases in its importance in adult speech processing.

### Phase Amplitude Coupling

Finally, we explored whether the phase of low-frequency oscillations act to modulate high-frequency amplitudes in similar ways in infants and adults. PAC was calculated using a composite complex-valued signal *z*(*t*, τ) combining the amplitude envelope *A*_1_(*t* + τ) of a high-frequency with the phase φ_2_(*t*) of a filtered low-frequency signal (Canolty et al., 2006; Özkurt and Schnitzler, 2011). The length of the resulting vector was used as a measure of coupling strength between two frequencies. As differences in low-frequency power have been shown to adversely affect the PAC calculation (Özkurt and Schnitzler, 2011; Aru et al., 2015; Jensen et al., 2016), a statistically normalized version of the modulation index (nMI) was calculated (see section “Materials and Methods”). For each participant low-frequency phases from 2 to 8 Hz (1 Hz steps) and high-frequency amplitudes from 15 to 45 Hz (5 Hz steps) were extracted from the EEG signal from each of the 128 electrode channels. For each of these PAC pairing steps, multiple nMI values were calculated per infant *via* a 5-s sliding window. The significant windows were identified if they exceeded the 95% confidence interval calculated from a surrogate data set made up of 200 statistical iterations of the same analysis window (see section “Materials and Methods” for full procedure).

In order to examine whether a similar pattern of PAC would be exhibited by adults and infants, a two-way repeated measures ANOVA was conducted with two levels, carrier phase and high-frequency amplitude. The aim was to examine whether the nMI values were significantly different when delta versus theta was the low-frequency carrier phase (low-frequency phase; levels of delta and theta) and when beta versus gamma was the high-frequency amplitude (high-frequency amplitude; beta or gamma).

The analysis showed a significant main effect of low-frequency phase [*F*(1, 20) = 16.499, *p* = 6.09 × 10^–4^], because theta phases (mean ± SEM, 3.530 ± 0.048) produced significantly higher coupling than delta phases (3.252 ± 0.046). There was no significant effect of high-frequency amplitude [*F*(1, 20) = 0.104, *p* = 0.751], and no significant interaction was observed between low-frequency phases and high-frequency amplitudes [*F*(1, 20) = 0.705, *p* = 0.411].

To further investigate the observed significant effect of phase, Bayesian related samples *t*-tests were conducted to explore the relative effect size within each PAC pairing. One way Bayesian repeated measures ANOVA’s were conducted separately comparing delta and theta phase coupling with either gamma or beta high-frequency amplitudes. There was strong evidence that theta/gamma coupling was greater than delta/gamma coupling (BF10 = 21.564), however, there was only anecdotal evidence that theta/beta coupling was greater than delta/beta coupling (BF10 = 1.896).

Overall, the PAC analyses suggest that theta is a more dominant carrier phase than delta when coupling with gamma amplitudes in the adult brain. This is different to infants, where both delta and theta show equal PAC (nMI) with gamma amplitudes when rhythmic speech was the input.

## Discussion

Here we replicated with adults a study of cortical tracking to sung speech originally conducted with infants aged 4, 7, and 11 months (Attaheri et al., 2022). Our aim was to explore whether neural responses to rhythmic inputs differ between infants and adults. Accordingly, the same PSD, mTRF stimulus reconstruction and PAC analyses were applied to EEG data recorded from adults, who had experienced the exact same paradigm used previously with infants. It was expected that developmental differences in both cortical tracking and PAC might be observed. Our methods were designed to enable us to distinguish between developmental effects and stimulus-driven phenomena.

Regarding cortical tracking, we found that the mTRF stimulus reconstruction data broadly matched the patterns found with infants (Attaheri et al., 2022), suggestive of no developmental differences. In both infants and adults, delta and theta EEG responses tracked the acoustic envelope of sung speech. Despite significant cortical tracking in both groups, the stimulus-induced PSD peaked at 1.25 Hz in the adult brain, whereas the infant PSD peaks were observed at 2.20 and 4.37 Hz. Only the infant PSD peaks corresponded to the prominent modulation spectrum peaks in the averaged nursery rhyme speech envelopes, suggestive of more stimulus-driven processing (Supplementary Figure 2). The lack of peaks at 2.20 and 4.37 Hz for adults may imply a more stimulus-driven response in the infant brain, which appears primarily to track the prominent modulation peaks in the nursery rhyme speech envelopes (Attaheri et al., 2022). It may also be due to the difference in the number of participants between the studies (∼60 infants vs 21 adults). Nevertheless, when the adult data were analyzed using the same center frequencies as the infant PSD peaks (2.20 and 4.37 Hz), significant increases in PSD power compared to resting state were observed for adults also.

The stimulus reconstruction analyses also showed a trend toward significant envelope tracking in the alpha band for the adults, an effect not found for infants. In the adult data, stimulus-induced PSD power also peaked at ∼8.54 and ∼10.04 Hz, both of these peaks occurring in the alpha band. Statistical analysis showed that only the peak at 8.54 Hz was significantly different to adult resting state data. Accordingly, the observed increase in low alpha power may be linked to the formation of additional linguistic processing mechanisms by the adults, for example related to comprehension or attention (Weisz et al., 2011). Bayesian analyses showed very strong evidence for delta band cortical tracking and decisive evidence for theta band cortical tracking. This may suggest that the relative importance of theta band tracking increases with development. Whilst most cortical tracking studies in adults report theta band tracking, there is a now growing body of literature reporting a strong role for delta band tracking in speech processing at lexically and semantically coherent word, phrase and sentence levels (see Ding and Simon, 2014; Kösem and van Wassenhove, 2017 for full review). This raises the possibility that delta band tracking has different functions for infants and adults, however, the current data do not enable any conclusions to be drawn. For our IDS stimuli, the stimulus reconstruction analyses showed that cortical delta and theta neural signals tracked the envelopes of the nursery rhyme stimuli, matching the findings with infants. The delta and theta band entrainment observed here appear to be mainly stimulus-driven effects, as they were also observed in pre-verbal infants. The main developmental difference observed was the relative increase in the strength of the theta band tracking in adults, which could be related to mechanisms important for comprehension and speech intelligibility (Ghitza, 2011). Nevertheless, both frequency bands showed significant cortical tracking in both populations.

**FIGURE 4:**
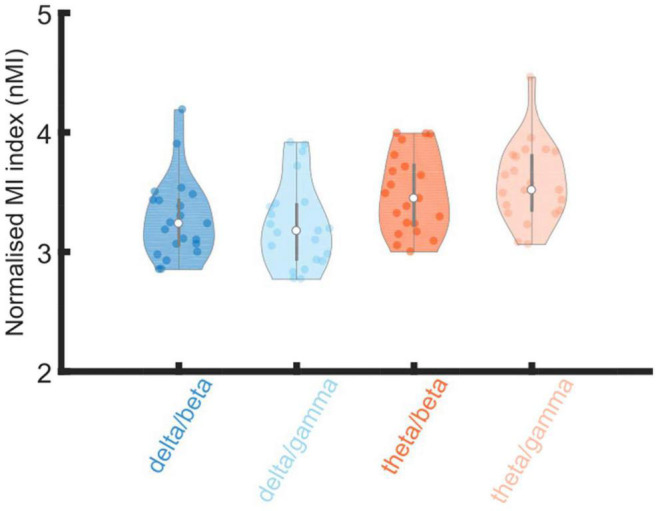
Violin plot of distributions of normalized modulation index (nMI) as measures of phase amplitude coupling (PAC). The PAC bands of interest are given on the X axis (delta/beta, delta/gamma, theta/beta and theta/gamma). Shades of blue denotes PAC pairs with delta as the low frequency phase and shades of orange denotes when theta was the low frequency phase. The nMIs were averaged together (from all significant analysis windows) for each participants data separately for each low frequency phase and high frequency amplitude pairing. The PAC pairing with the maximum nMI, per participant, from within the pre-defined frequency bands of interest; delta 2–4 Hz, theta 4–8 Hz, beta 15–30 Hz and gamma 30–45 Hz), were included in the grand average violin plot.

The beta and gamma high frequency amplitudes utilized here (15–30 Hz and 30–45 Hz) showed significant coupling to the delta (2–4 Hz) and theta (4–8 Hz) low frequency phases in both infants and adults. However, clear developmental differences in the pattern of phase amplitude coupling were observed. Infants showed significant differences in the high frequency amplitudes used but not in the low frequency phases. Adults showed significant differences in the low frequency phases used but not the high frequency amplitudes. The Bayesian analyses indicated strong evidence that theta/gamma coupling was greater than delta/gamma coupling for adults. This may also relate to the TEMPO model proposed by Ghitza (2011), who has suggested that specific theta-gamma couplings are required when speech is processed for meaning. This pattern differed from prior findings with infants, as the infant brain showed similar levels of PAC to gamma when either delta or theta was the low frequency carrier (the interested reader is invited to compare Attaheri et al., 2022; Figure 4, with Figure 4 here). However, the infant brain did show a significantly higher nMI when gamma was the high frequency amplitude rather than beta. Given previous adult data (Hyafil, 2017), beta was also expected to be less important than gamma for adult PAC. In the adult data, nMI values were greater when gamma rather than beta was coupling to theta phases, with evidence that theta/gamma coupling was stronger than delta/gamma coupling. Overall, the PAC analyses suggest that theta is a more dominant carrier phase than delta when coupling with high frequency amplitudes in the adult brain. This is different to PAC in infants, where both delta and theta showed equal normalized PAC to gamma amplitudes when sung speech was the input.

The current work has a number of limitations. Ideally, children at different ages (i.e., after 11 months) as well as adults should experience the sung speech paradigm while EEG is recorded. This would provide a better assessment of developmental trajectories. The number of adult participants (21) utilized here was also much smaller than the number of infants studied by Attaheri et al. (2022) (∼60 each at ages 4, 7, and 11 months), hence the number of participants could be increased. It could also be interesting to compare adults with and without musical training using the same materials, in order to see whether the use of sung speech reveals any effects of musical expertise on adult cortical tracking. Finally, it could be interesting to record EEG while adults listen to sung nursery materials in unknown languages. This would allow the effects of familiarity and comprehension of the sung speech to be disentangled from the fact that the speech is sung, equating adults and infants for non-maturational factors related to differences in linguistic expertise.

In conclusion, by matching exactly the experimental paradigm and the analysis methods used to investigate cortical tracking of sung speech in infants versus adults, we have revealed more developmental similarities than developmental differences in entrainment. The main differences related to increasing prominence of theta-band mechanisms with age and language experience. While both delta and theta band tracking were observed in the adult brain, the Bayesian analyses showed “decisive” evidence for theta band cortical tracking but “very strong” evidence for delta band cortical tracking. There was also “moderate” evidence for alpha band tracking. Further, there was strong evidence that theta/gamma coupling was stronger than delta/gamma coupling for adults. In the case of infants, both delta and theta showed equally strong coupling to gamma. Whilst we can only speculate regarding the mechanisms underpinning our observed phase/amplitude alignments, previous literature suggests the alignments may be caused by both modulatory and evoked effects (Lakatos et al., 2009; Gross et al., 2013). The increasing role for theta signals in speech processing by adults could reflect many factors, including neural maturation, increased language experience, better language comprehension, and even learning written language, as phase locking in the theta band to rhythmic speech is known to increase in children in line with their reading ability (Power et al., 2012).

## Data Availability Statement

The datasets presented in this study can be found in online repositories. The names of the repository/repositories and accession number(s) can be found below: https://osf.io/s9ezd/.

## Ethics Statement

The study was reviewed by the Psychology Research Ethics Committee of the University of Cambridge, and after a detailed explanation of the study, written consent was given by each participant.

## Author Contributions

AA: EEG paradigm development, EEG preprocessing, investigation—data curation, formal analysis—design, creation and implementation of analysis, and writing—original draft. DP and AP: predominant data collection. ÁC: data curation—writing—review and editing. GD: formal analysis, writing—review and editing. SR: writing—review and editing. PB: EEG paradigm development and investigation. NM: investigation—data curation. SF: Analysis: modulation spectrum analysis. HO-S: investigation—data curation. UG: conceptualization—methodology, funding acquisition, supervision, project administration and writing—original draft. All authors contributed to the article and approved the submitted version.

## Conflict of Interest

The authors declare that the research was conducted in the absence of any commercial or financial relationships that could be construed as a potential conflict of interest.

## Publisher’s Note

All claims expressed in this article are solely those of the authors and do not necessarily represent those of their affiliated organizations, or those of the publisher, the editors and the reviewers. Any product that may be evaluated in this article, or claim that may be made by its manufacturer, is not guaranteed or endorsed by the publisher.

## Footnotes

1
http://audition.ens.fr/adc/NoiseTools/
