# Supplementary material for: Cortical Tracking of Sung Speech in Adults vs Infants: A Developmental Analysis
Source: Front Neurosci. 2022 Apr 12;16:842447. doi: 10.3389/fnins.2022.842447 (PMC9039340; doi:10.3389/fnins.2022.842447)
Supplement: Supplementary file 1 [file Data_Sheet_1.docx]

## Supplement

**1 PSD across recording conditions**


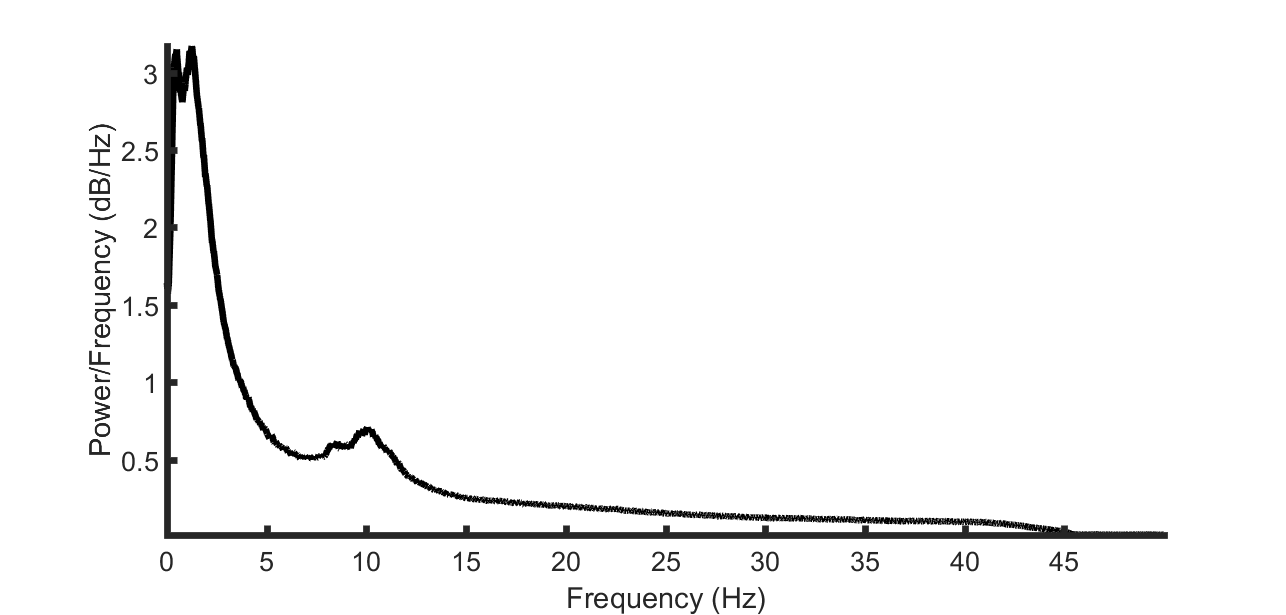


Supplementary Figure S1, Grand average PSD power across stimulus and resting state recordings. The grand average plot displays peaks at 1.25Hz, 8.54Hz and 10.04Hz which were used as center peak values in the PSD analysis.

**2 Clean_ASR EEGLab function**

Artifact Subspace Reconstruction (ASR), EEGLab toolbox (Delorme & Makeig, 2004), is an automated artifact rejection function. It uses principle component analysis (PCA) to ensure no period of the data signal has abnormally strong power. First the algorithm finds the cleanest portion of the data to use as the calibration data, and the first statistics are computed. Next, a 0.5 second sliding window PCA (with 50% window) is performed across all the channels to identify bad PCs. The algorithm next finds the subspaces in which the activity that is more than 5 standard deviations away from the calibration data. The high variance subspaces (i.e. group of channels associated with the ‘bad’ PC’s) are then reconstructed using a mixing matrix that was calculated on the clean data.

**3 Modulation Spectrum of nursery rhyme sound files**

All analysis was computed using MATLAB 2016a. The modulation spectrum extraction was based on an approach described by Plomp, et al., (1) and developed by Leong, et al. (2). First, the sound files were down sampled to 14.7 kHz and then band-pass filtered using a series of adjacent FIR filters, into five bands: 100-300 Hz, 300-700 Hz, 700-1750 Hz, 1750-3900 Hz, and 3900- 7250 Hz. Next, the Hilbert envelope was extracted from each of the five sub-band signals. The five envelopes were down sampled to 1050 Hz then filtered through a modulation filter bank. This modulation filter bank comprised 24 channels logarithmically spaced between 0.9-40 Hz. In Figure S2, the RMS difference power was averaged across 18 nursery rhymes and determined for each spectral band. In Figure S3, for one nursery rhyme, the RMS power in each spectral band was divided by the overall RMS power, revealing the relative amount of energy in each band. In Figure S4, the “all band averages” from each of the 18 individual nursery rhymes are depicted together, as denoted by the thin multicolored lines. The grand average of these 18 individual “all band averages” and STD are denoted by the black line and grey shading respectively. Modulation filter bank corner frequencies were taken as [0.93; 1.09; 1.27; 1.49; 1.74; 2.03; 2.38; 2.78; 3.25; 3.80; 4.45; 5.20; 6.08; 7.11; 8.32; 9.72; 11.38; 13.30; 15.56; 18.20; 21.28; 24.89; 29.11; 34.04; 39.81]. Clear peaks in modulation power can be observed at ~2.18 and ~4.4Hz.


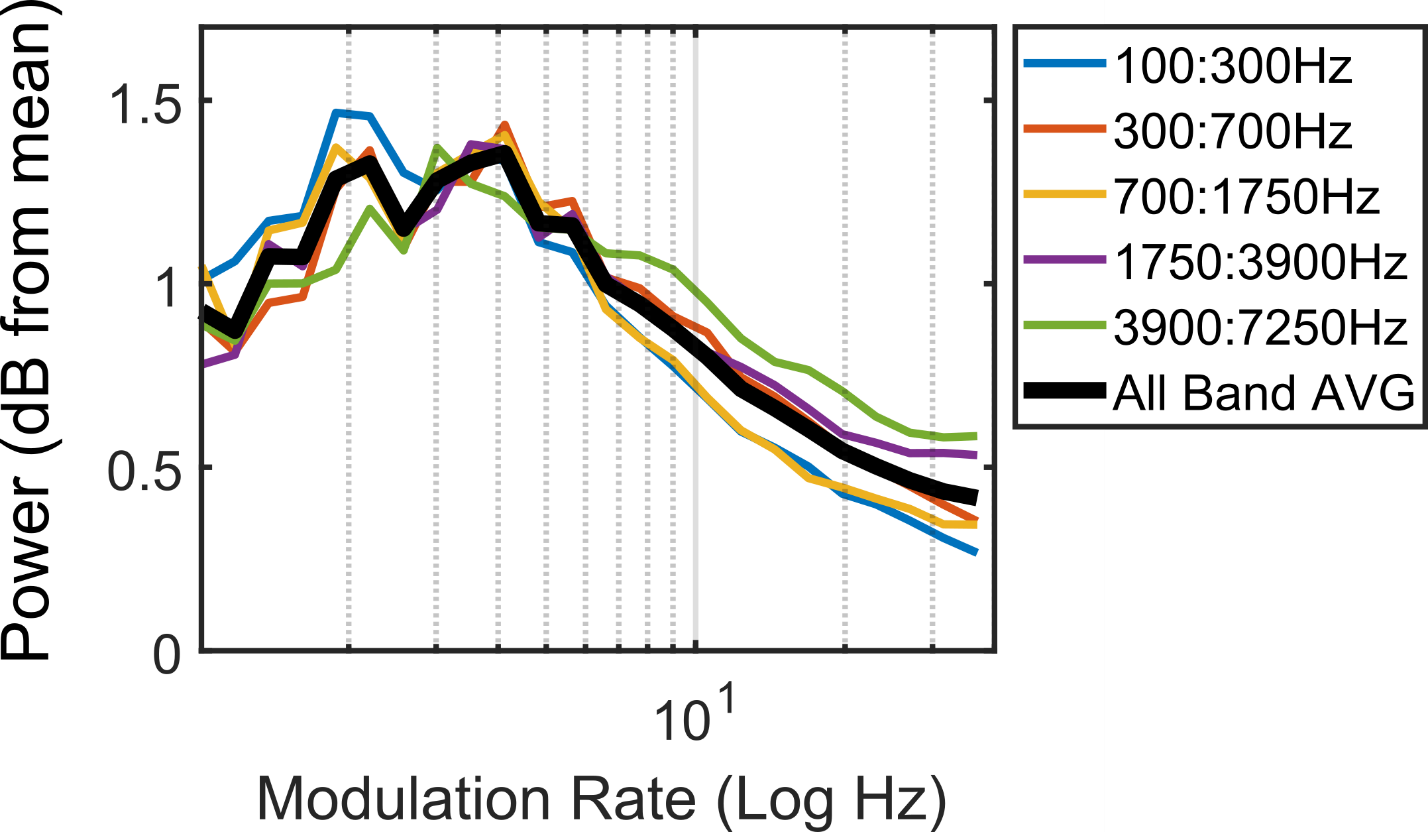


Supplementary Figure S2, Average modulation spectrum of all the Nursery rhyme stimuli.


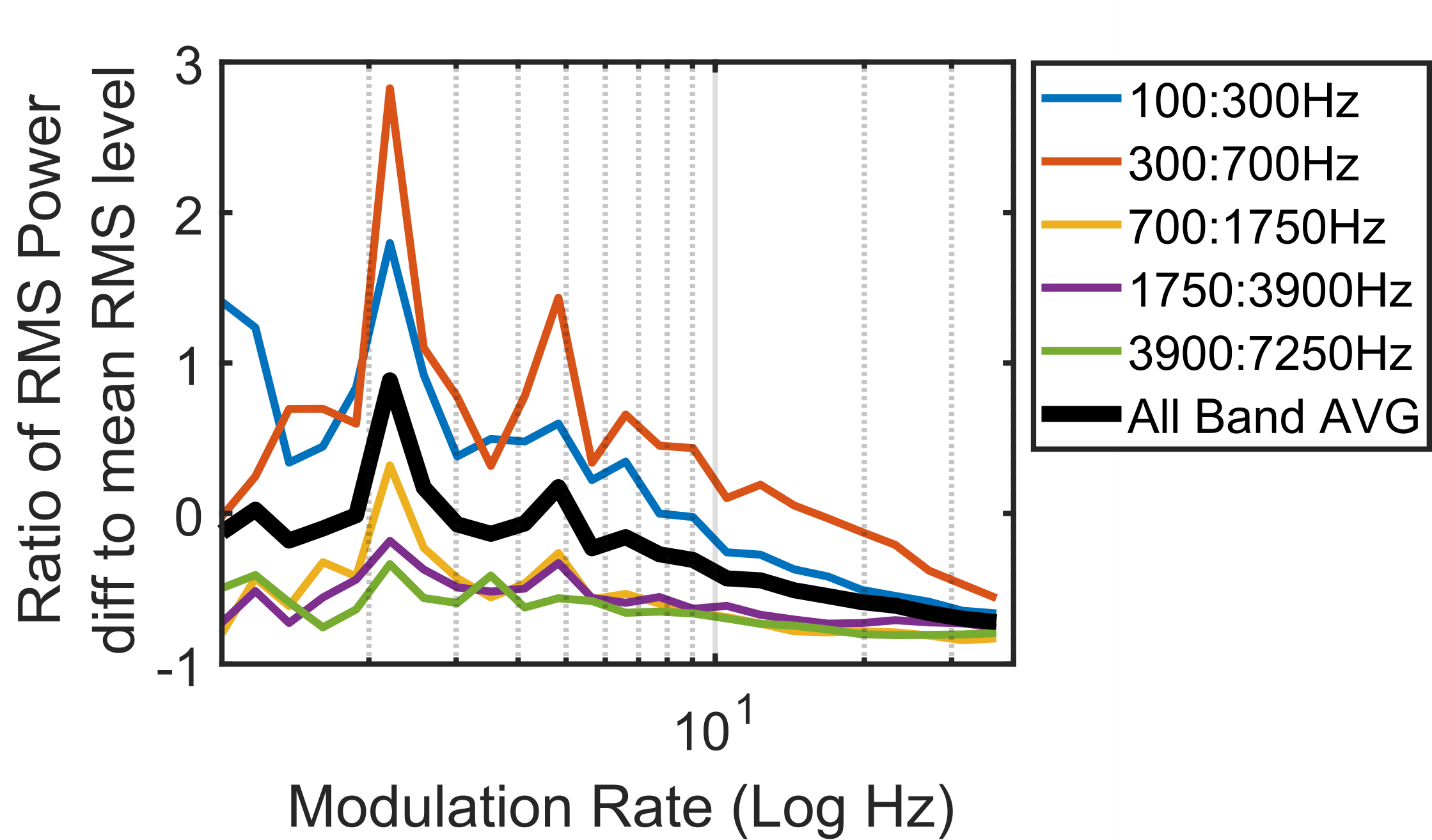


Supplementary Figure S3, Example modulation spectrum of one the nursery rhyme stimuli (‘Simple Simon’). RMS power in each spectral band was derived by the overall RMS power.

**
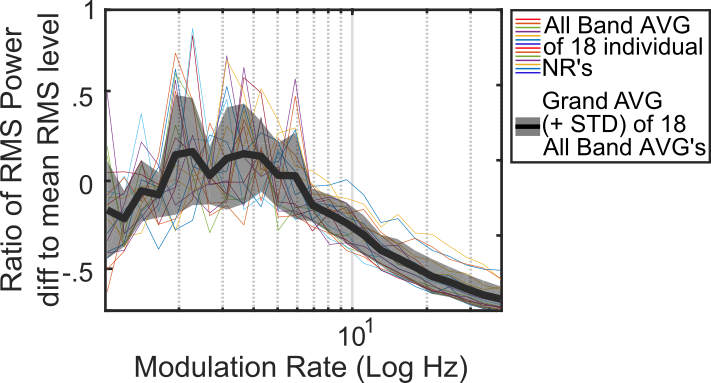
**

Supplementary Figure S4, Grand average of the 18 individual nursery rhymes “All Band AVG” Modulation spectrums, with the “All Band AVG” depicted for each individual rhyme by the separate multicolored lines. The grand average of the 18 individual “All Band AVG”, and their standard deviations (STD), are denoted by the black line and grey shading respectively.

**4 Supplementary references**

1. Plomp, R. Perception of speech as a modulated signal. Proceedings of the 10th International Congress of Phonetic Sciences. (Utrecht), 29–40. (1983).
2. Leong, V. Prosodic rhythm in the speech amplitude envelope: Amplitude modulation phase hierarchies (AMPHs) and AMPH models. Doctoral dissertation, University of Cambridge. (2012).
